# Study on the Effect of Employees’ Perceived Organizational Support, Psychological Ownership, and Turnover Intention: A Case of China’s Employee

**DOI:** 10.3390/ijerph19106016

**Published:** 2022-05-15

**Authors:** Jianwan Jing, Jinzhe Yan

**Affiliations:** 1 Department of Business, Gachon University, Seongnam 13120, Korea; jingjianwan0305@naver.com; 2 School of Business, Gachon University, Seongnam 13120, Korea

**Keywords:** Chinese enterprises, perceived organizational support, turnover intention

## Abstract

In the context of severe turnover, taking measures to enhance core employee management, prevent the turnover of talents, and improve employees’ sense of belonging and responsibility to the firm can become a non-negligible problem in human resource management. Considering Chinese enterprises as the research background, this study starts with the related theories of organizational support, psychological ownership, and turnover intention to explore the impact of organizational support on psychological ownership and its dimensions (self-efficacy, taking responsibility, a sense of belonging, and self-identification), the effect of each dimension of psychological ownership on turnover intention, and the relationship between organizational support and turnover intention, as well as verifies the mediating role of psychological ownership. The main findings show that (1) perceived organizational support positively affects psychological ownership; (2) psychological ownership negatively affects turnover intention; (3) perceived organizational support negatively influences turnover intention; and (4) psychological ownership mediates the relationship between perceived organizational support and turnover intention. The study results contribute to the relevant literature and guide human resource practice.

## 1. Introduction

Since Pierce et al. [1] first proposed the concept of psychological ownership (PO), studies on psychological ownership in organizational behavior have steadily increased. Organizational behavior scholars have gradually recognized that psychological ownership plays an important role in talent management in organizations, especially nowadays, and that enterprise employees’ pursuits of novelty and information and their requirements for self-fulfillment have prompted a change in their perception of the profession as well. This psychological change contradicts the unchanged management style developed in the long-term development of enterprises, which makes employee turnover a common phenomenon in modern economic society.

In the mid-1980s, Eisenberger et al. [2] proposed the Organizational Support Theory based on the social exchange theory and the principle of reciprocity, that is, the willingness of employees to stay in the organization and contribute their strength largely depends on perceived organizational support (POS). In other words, organizational support to employees is the first step before employees commit to the organization [3]. Cropanzano et al. [4] showed that a high sense of organizational support could enhance employees’ self-identity at work and generate the “master” spirit. Moreover, organizational psychological ownership manifests employee ownership consciousness, whereby employees perceive the organization as a “possession”, thus manifesting a psychological state in which the organization of “my goods” holds oneself close to the organization. At the same time, organizational support can directly lead to employees’ emotional dependence on the organization to be a “home” which can lead employees to think that the organization is “mine” or “ours”, resulting in a sense of belonging and responsibility [5]. Compared with employees from a Western background, Chinese employees are more likely to have similar needs and expectations for the organization as “home”, that is, the conditions for psychological ownership. Wagner et al. [6] stated that employee organizational psychological ownership is a good predictor of employee attitudes and work behaviors, such that when an organization provides support and resources to employees, they experience positive feelings about the organization. This allows employees to perceive their organization as a “home” and themselves as part of this “home”, working voluntarily as an organizational effort. If people outside pass bad comments on their company, they will be dissatisfied and actively maintain their organization’s image. Conversely, if the enterprise excessively burdens the employees while employees face family conflicts or work problems and if the enterprise fails to provide timely help and support, the employees will develop an intention to leave, which leads to an increase in turnover and unstable organizational structure [7].

The turnover phenomenon is a waste of resources invested by the company in early training. Employee turnover means the loss of human resources, which is not conducive to the enterprise’s long-term development and implementation of the strategy. Loss of talent is a massive loss to a company. The loss of human resources leads to the loss of the company’s resources, culture, technology, and philosophy. Moreover, intercorporate competition is increasing in all countries. Various scholars conducted turnover intention related research [8,9,10,11], aiming to reduce resource waste and improve the effectiveness of human resources management. Yang, Pu, and Guan [8] documented that entrepreneurial leadership affects employees’ turnover intention. Zhang, Meng, Yang, and Liu [9] examined job satisfaction and work engagement negatively associated with turnover intention. Moreover, professional identity mediates the relationship between job satisfaction, work engagement, and turnover intention.

Therefore, human capital, an essential part of the core competitiveness of each firm, is a non-negligible component. Therefore, in human resources management (HRM), adopting measures to strengthen core employee management and prevent the loss of talents; strengthen the psychological ownership of organizational members; and enhance employee loyalty to the firm, feelings of belonging, and responsibility has become non-negligible.

Therefore, this study starts with the related theory of organizational support, psychological ownership, and turnover intention to explore the effect of the perception of organizational support on psychological ownership and its various dimensions (self-efficacy, accepting responsibility); the effect of each dimension of psychological ownership on turnover intention; and the relationship between the perception of organizational support and turnover intention to verify the mediating role of psychological ownership. Then, through a survey, this study establishes the relationship model between variables, empirically verifies the impact of the proposed relationship on the hypothesis, and further refines the related theory to provide a valuable reference for Chinese firms to improve HRM and reduce turnover rate.

First, the theoretical implication lies in the following. Academicians have actively discussed the influence of employees’ active departure. Turnover intention is analyzed through personal, employment, and environmental factors, but turnover is often a complex psychological process; not one factor alone leads to employee departure, and no single measure can effectively control employee departure. Studies have focused on the factors that are relevant to turnover. However, the relationship between psychological factors and turnover intention is understudied, and few studies have combined organizational, psychological, and turnover intention. Therefore, this study addresses this deficiency by adding psychological ownership variables between organizational support and turnover intention, which open up a new path for the study of the turnover intention model. This is an attempt to study the influence of organizational support on turnover intention in Chinese enterprises with psychological ownership, as an intermediary variable by adding psychological research variables. Second, this study has three practical implications. The first is to enhance the understanding of the actual situation of human resources in Chinese enterprises through a questionnaire to understand the employees’ organizational support in Chinese enterprises, the current situation of turnover intention, and its related relationship, as well as to deepen the understanding of the mediating role of psychological ownership in it, which will improve our understanding surrounding the perception of the management situation of Chinese enterprises. The second is to guide the enterprise to effectively motivate employees, deepen the understanding and support of employees, and promote the relationship between enterprises and employees. To enhance employees’ awareness of organizational support involves helping employees maximize their awareness of the enterprise’s support to correct their working attitudes and behavior and make greater efforts to pay for the enterprise. This study can help enterprises better understand the significance of providing effective organizational support to enhance employee’s sense of belonging and loyalty toward the organization. The third is to strengthen the importance of businesses place on employees’ psychological aspects. Psychological ownership is an important psychological state of employees in their work, which directly impacts their work behavior. Therefore, understanding and attaching importance to employees’ psychological ownership can improve employee management in enterprises. Employees treat the firm with a sense of ownership, effectively reducing the employee’s intention to leave, preventing the loss of talent, and improving firm cohesion.

## 2. Literature Review and Hypothesis Development

### 2.1. Perceived Organizational Support

Eisenberger proposed the concepts of the organizational support theory (OST) and POS based on the social exchange theory and the reciprocity principle, in which organizational support is the core of the organizational support theory. Eisenberger, Huntington, Hutchison, and Sowa [2] stated that the primary source of the organization–employee relationship is mutual needs and expectations and that employees are willing to work for the organization for the compensation received. Therefore, effective motivation can only be generated by knowing and meeting the employees’ needs. Thus, organizational support is defined as an organization’s assessment of its employees’ contributions and focus on their well-being, resulting in a holistic and comprehensive understanding of organizational support [2]. McMillan [12] extended and supplemented Eisenberger’s OST with a large body of empirical research. McMillan [12] argued that without substantial instrumental support, employees have no antecedents and foundations for accomplishing their job and are far less guaranteed to complete the job efficiently. In their research on expatriate organizational support, Kraimer et al. [13] grouped organizational support into three parts: developmental, financial, and adaptive support.

In the variable study on organizational support, Rhoades and Eisenberger [3] highlighted that the pre-dependent variables that predict organizational support include organizational equity [4,14,15], supervisor support [16], organizational treatment, work environment [17], and personal characteristics of employees. Compared with the predictive effect, organizational factors such as organizational fairness have a strong predictive effect on organizational support, while employees’ individual characteristics have a weak influence [3]. Research on organizational support sense outcome variables has largely included job performance [18,19,20], organizational citizenship behavior [21,22], organizational commitment [3,4,5], turnover behavior [15,23], and job satisfaction [24]. In recent years, organizational support has also been used as an intermediary variable [15,20,25,26,27,28].

### 2.2. Organizational Support and Psychological Ownership

According to the Employee Stock Ownership Program, Pierce, Rubenfeld, and Morgan [1] proposed the concept of psychological ownership for formal ownership. They suggested that employee shareholdings would not affect corporate performance and employee job attitude through formal ownership and would only positively affect corporate performance and employee job attitude/behavior through psychological ownership. Dirks et al. [29] argued that psychological ownership, the target of individual generation, is a psychological state belonging to the individual. Parker et al. [30] regarded psychological ownership as the responsibility generated toward the target objects. Pierce et al. [31] extended the concept of psychological ownership and defined psychological ownership as an ideology that produces a psychological state of “mine” or “ours” by individuals toward a target. In business organizations, Avey et al. [32] believed that employees’ psychological ownership refers to the multidimensional structure of self-efficiency, responsibility, belonging, and self-identity, which regards the part of the organization or work as belonging to them and reflects individuals’ awareness, thoughts, and beliefs about the organization.

Pierce, Kostova, and Dirks [31] proposed that three pathways enhance individual psychological ownership. First, the organization should give employees more autonomous job opportunities that promote a sense of employee control over the work being performed and, ultimately, improve the organization’s psychological ownership. Second, the extent to which an employee knows about the job and organization is positively related to the degree of psychological ownership generated by the organization. Third, the more the employees invest in the work and organization, the higher the psychological ownership. When used in organizational situations, psychological ownership can be divided into job-based and organization-based psychological ownership [33]. Job-based psychological ownership refers to speaking about employee occupancies arising from the job or part of the job they have undertaken. Organization-based psychological ownership refers to employee occupancies regarding the organization. This study uses the concept of organization-based psychological ownership, which means that when employees are aware of the organization’s possession, they also have a psychological state of interest in sharing with the organization.

Concerning psychological ownership, this study reviewed related literature. The pre-dependent variables of psychological ownership are formal ownership [1], work autonomy [30,34], work control [1,35,36], organizational fairness of non-family employees in family enterprises [37], and interpersonal factors [38,39]. Outcome variables of psychological ownership included job satisfaction [34], organizational commitment [40,41], TI [39], organizational citizenship behavior, out-of-role behavior [6,39,40,42], constructive dereliction [43], territorial behavior [44], and employee attitudes toward “organizational change” [31,40,45]. Of the studies with psychological ownership as a mediating variable, the Sieger et al. [46] findings showed a mediating role between the perception of distributive fairness and affective commitment, and Liu et al. [47] argued that participation in decision-making, self-management team climate, organizational self-esteem, and effective commitment has a mediating role. Additionally, Knapp et al. [48] showed that the relationship between organizational identification and turnover intention is completely mediated by the perception of insider status and psychological ownership.

Studies on the relationship between the perception of organizational support and psychological ownership have indicated that the perception of organizational support is beneficial for promoting the production of employees’ psychological ownership of the organization and its multidimensional structure (self-efficacy, taking responsibility, a sense of belonging, and self-identification). Eisenberger et al. [49] and Wayne, Shore, and Liden [23] argued that if an employee feels that organizational support comes from voluntary actions within the organization, rather than driven by external factors, the employee perceives that the organizational support results from the organization’s “true positive treatment” of the employee, thus enhancing organizational commitment and generating psychological motivation to reward the organization. O’driscoll, Pierce, and Coghlan [42] highlighted that providing employees with an autonomous working environment is conducive to enhancing the psychological ownership of their work and organization, thus actively avoiding negative work attitudes and behaviors. Hameed, Hameed et al. [50] demonstrated that high levels of organizational support can enhance the relationship between psychological ownership and knowledge sharing behavior.

Regarding the relationship between organizational support and self-efficacy, Armeli et al. [51] stated that when employees feel that the organization’s supports them, they will think that the organization respects and attaches importance to them, and meets the self-efficacy of “being recognized” and “being praised”. Regarding perceived responsibility for organizational support, scholars such as Avey, Avolio, Crossley, and Luthans [39] argued that the exchange between the organization and employee is beneficial for promoting the employee’s feelings of responsibility in return toward the organization, which, in turn, forms psychological ownership, a relationship known as one of the formation paths of employee organizational psychological ownership. Because psychological ownership has the characteristics of responsibility and belonging, employees will change their attitudes toward the organization, and employees who emotionally depend on the organization will be happy to participate in its work [52]. A higher sense of organizational support enables employees to meet their social-emotional needs, thereby strengthening their “intimate relationship” with the organization and increasing their “sense of responsibility” and “emotional dependence” [20,22].

Organizational support meets emotional needs such as employee attribution, that is, when an organization respects its employees, values their results, and cares about their lives, it leads to emotional attribution of the employees [2,51]. Stinglhamber and Vandenberghe [5] found that organizational support contributes to employees’ sense of organizational responsibility, belonging, and positive emotions.

Daneji and Bambale [53] considered psychological ownership as innately possessed and referred to the conscious feeling of ownership for a certain thing, place, or anything. Individuals perceive the object of ownership as an extension of themselves and feel a sense of responsibility toward the object of ownership. Higher organizational support promotes employee self-identity at work and generates a sense of ownership [4,43].

Based on previous research on the relationship between the perception of organizational support and psychological ownership, this study believes that the perception of organizational support is beneficial for promoting the generation of employees’ psychological ownership of the organization and its multidimensional structure (self-efficacy, taking responsibility, a sense of belonging, and self-identification). Therefore, the following hypotheses are proposed:

**Hypothesis** **1** **(H1).** *Organizational support has a positive impact on psychological ownership*.

H_1-1_–H_1-4_: Organizational support has a positive effect on several components of psychological ownership, including self-efficiency (H_1-1_), accountability (H_1-2_), a sense of belonging (H_1-3_), and self-identity (H_1-4_), respectively.

### 2.3. Perceived Organizational Support and Turnover Intention

Mobley et al. [54] defined turnover intention as an employee who, after working for some time in the organization, becomes dissatisfied with the organization or work, leaves the existing job, and seeks comprehensive performance and ideas for other jobs. Currently, the concepts can be divided into broad and narrow senses in which the broad sense represents the transfer of employees between regions, organizations, industries, and positions. In the narrow sense of the concept of turnover, Ellett et al. [55] categorized departure as voluntary, involuntary, and unavoidable. This study uses voluntary termination in a narrow sense of the concept of termination.

According to social exchange theory, when the organization values the benefits and treatment of organizational members and recognizes employees’ contributions and achievements, employee enthusiasm for work is stimulated, and job satisfaction increases. Furthermore, because the psychological attainment of satisfaction and willingness to work for the organization also increases when perceived support from the organization, it further decreases employees’ turnover intention. Specifically, when employees feel that the organization is concerned about their actual interests, they have positive motivations and behaviors toward the organization, such as a sense of responsibility or loyalty [3]. Conversely, if the enterprise overburdens the employees, or if the enterprise is unable to give timely help and support when employees are facing family conflicts or work problems, they will have turnover intention, which increases the number of employees tending to leave the enterprise and an unstable organizational structure [7].

Eisenberger et al. [56] suggested that the stronger an employee’s sense of organizational support, the less likely they are to find new jobs and leave the organization. Several studies have confirmed that organizational support is positively related to organizational commitment and that organizational commitment affects employee turnover intention [23,57,58,59]. Lazarova and Caligiuri [60] demonstrated that organizational support is negatively correlated with turnover intention in the study of returnees. Jawahar and Hemmasi [61] showed that a sense of organizational support directly impacts female employees’ turnover intention, and the enhancement of organizational support can enhance women employees’ sense of responsibility and trust. Srivastava and Agrawal [62] showed that high organizational support reduces the strength of the relationship between job burnout and change resistance, and turnover intention. Thus, Hypothesis 2 is proposed:

**Hypothesis** **2** **(H2).** *Organizational support has a negative impact on turnover intention.*

### 2.4. PO and TI

With the introduction of the concept of psychological capital into foreign scholars’ study of human resources, many scholars also realized that psychological ownership plays a crucial role in an organization’s talent management, especially on the turnover tendency. Furthermore, Larson and Luthans’ [63] study on psychological factors and employee behavior showed that psychological capital has a significant impact on employees’ work attitudes, which is much larger than human and social capital and has a predictive effect on employees’ work attitudes. Through the combined induction of various studies, the antecedent variables of turnover intention are found; thus, the influencing factors have diverse and complicated characteristics, which can be grouped into four types: demographic variables of employees, such as gender, age, marital history, and education level [64,65,66]; organizational external environment variables, such as social and economic situation, labor market situation, and external job opportunities; work-related variables within the organization [65], such as job benefits, promotion opportunities, job characteristics, and job stability [55,67,68]; and employee job relationship variables, such as organizational support, organizational satisfaction, and organizational commitment [65,67,69,70,71,72,73].

When employees have low psychological ownership, they are emotionally separated from the organization and unwilling to work autonomously, generating turnover intention [39,43]. Concerning the responsibility of psychological ownership for specific objectives, Wagner, Parker, and Christiansen [6] believed that employees with psychological ownership see themselves as part of the organization and are willing to protect the organization and will not leave the organization. Olckers and Plessis [74] believed that employee psychological ownership can positively impact an organization’s effectiveness and retain talent for the company. Becker et al. [75] found that psychological ownership was more predictive of employees’ attitudes and behaviors and elicited a stronger sense of occupancies among employees. Individuals are willing to invest more time and effort into the organization, actively defend collective interests, consider issues from an organizational perspective, and do not generate turnover.

Based on previous literature findings, the following hypotheses were proposed:

**Hypothesis** **3** **(H3).** *Psychological ownership has a negative impact on turnover intention.*

H_3-1_–H_3-4_: Self-efficiency (H_3-1_), accountability (H_3-2_), sense of belonging (H_3-3_), and self-identity (H_3-4_) are components of psychological ownership which each have a negative effect on turnover intention.

### 2.5. Mediation of Psychological Ownership

Shore and Wayne [22] argued that employees’ feelings of organizational support are mainly derived from the perceptions of support, such as emotions and resources given to the organization. This perception directly affects changes in employees’ psychological state and behavior toward the organization, so the changes in psychological state can be a mediating variable between the feelings of organizational support and the variables of employee behavior.

O’Driscoll, Pierce, and Coghlan [42] highlighted that when employees perceive the opportunity of support, help, and development provided by the organization, they also understand that peer needs to adapt to their work style, form a good network of interpersonal relationships, and deepen the attachment of members toward the organization, thereby strengthening the influence of psychological ownership and weakening employees’ turnover intention. Additionally, through the previous discussion on the relationship between organizational support, the four dimensions of psychological ownership (self-efficacy, accountability, sense of belonging, and self-identity), and turnover intention, this study observed that organizational support has an impact on all dimensions of employees’ psychological ownership (self-efficacy, accountability, sense of belonging, and self-identity). However, each dimension of psychological ownership (self-efficacy, accountability, sense of belonging, and self-identity) has a significant predictive effect on turnover tendency, while organizational support impacts employees’ turnover intention. Therefore, it can be inferred that each dimension of psychological ownership mediates the relationship between organizational support and turnover intention. Therefore, H_4_ was derived:

**Hypothesis** **4** **(H4).** *Psychological ownership mediates the relationship between organizational support and turnover intention.*

H_4-1_–H_4-4_: self-efficiency (H_4-1_), accountability (H_4-2_), a sense of belonging (H_4-3_), and self-identity (H_4-4_) are components of psychological ownership which each mediate the relationship between organizational support and turnover intention.

**Figure 1:**
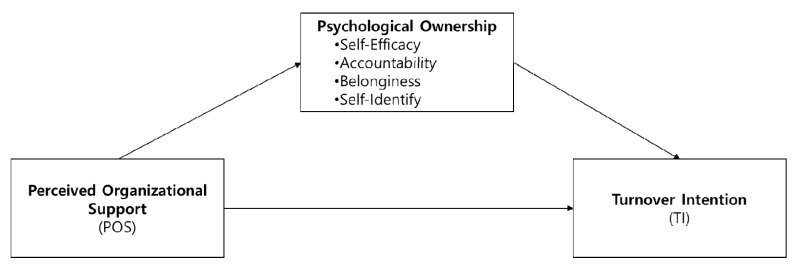
Research model.

Figure 1 presented he conceptual research model. 

## 3. Research Methodology

### 3.1. Measurement Design

The variable measurement scales were adopted based on previous empirical studies. All the measurement scales are well developed to test validity and reliability. Perceived organizational support was constructed following Wayne, Shore, Bommer, and Tetrick [17]. To measure psychosocial ownership, this study adopted the method proposed by Avey et al. [39], including four dimensions: self-efficiency, accountability, sense of belonging, and self-identity. Moreover, the scales proposed by Mobley, Horner, and Hollingsworth [55] were accessed to examine turnover intention. All items used in this study are presented in Table 1. This study used a five-point Likert scale ranging from 1 (“strongly disagree”) to 5 (“strongly agree”). The survey includes personal information (gender, age, education, position level, work experience) and organizational information (business size and industry).

### 3.2. Data Collection and Samples

This study collected data on an online survey platform, Sojump.com, the largest online survey platform in China. This study purchased paid samples service from sojump.com, and the sample services randomly distributed questionnaires to their qualified panels, who were employed by China’s Firms. Researchers did not involve or administrate the whole process of data collecting. At the beginning of the survey, all respondents are announced that they have the right to refuse to answer the survey if they feel negative emotions or terminate the survey for any reason. This study also promised that the survey does not contain identifying information or other private information. At the end of the survey, this study asked respondents to report if they felt negative emotions. After finishing the survey, all the respondents received monetary credits from the sample service of Sojump.

This study ensured the voluntary respondents’ anonymity and the elaboration of findings. Furthermore, national legal requirements did not require additional research ethical approval because this study does not collect identifier information and private information. It is stated that this study met the requirements of the Helsinki Declaration.

A total of 344 valid response questionnaires were collected, which is over the minimum requirement (five samples for each indicator) to conduct statistical analysis. Table 2 presents the demographic description of the sample.

## 4. Empirical Results

### 4.1. Descriptive Statistics, Reliability, and Correlation Analysis

The mean and standard deviation of descriptive statistics are shown in Table 3. The result of the reliability analysis shows that Cronbach’s α of all variables and latitudes is greater than 0.7, which indicates that the questionnaire has good reliability. Relevant analysis results show that organizational support, self-efficiency, responsibility, sense of belonging, self-identity, psychological ownership, and turnover intention are related. The research models and assumptions are initially verified.

### 4.2. Validity Analysis

Principal component analysis showed that the KMO value of the overall questionnaire was 0.878, which is greater than 0.7. The χ^2^ value of Bartlett’s test of sphericity was 4643.308, with a significance level of less than 0.001, indicating good construct validity of the questionnaire data and the presence of common factors, which were suitable for factor analysis. The overall explained variance reached 69.582%, and six eigenvalues were greater than 1(See Table 4). Moreover, the subject items of each variable and latitude were all loaded under that variable, which indicated a good construct validity.

### 4.3. Regression Analysis and Hypothesis Testing

This study performed regression analysis for hypothesis testing(results see Table 5). The empirical results reveal that model 5 shows that perceived organization support has a significant positive effect on psychological ownership (b = 0.452, *p*-value < 0.001), supporting H_1_; that perceived organization support has a significant positive effect on self-effectiveness (b = 0.357, *p*-value < 0.001), supporting H_1-1_; that perceived organization support has a significant positive impact on accountability (b = 0.295, *p*-value < 0.001), supporting H_1-2_; that perceived organization support had a significant positive impact on sense of belonging (b = 0.356, *p*-value < 0.001), supporting H_1-3_; and that perceived organization support has a significant positive effect on self-identity (b = 0.165, *p*-value < 0.01), supporting H_1-4_. Moreover, the regression results show that organizational support has a negative impact on turnover intention (b = −0.257, *p*-value < 0.001), supporting H_2_.

The regression results show that psychological ownership (b = −0.421, *p*-value < 0.001), self-efficacy (b = −0.327, *p*-value < 0.001), accountability (b = −0.268, *p*-value < 0.001), ownership (b = −0.289, *p*-value < 0.001), and self-identity (b = − 0.177, *p*-value < 0.001) has a negative effect on turnover intention, thus supporting H_2_, H_2-1_, H_2-2_, H_2-3_, and H_2-4_.

Mediation Test. As the effects of organizational support on psychological ownership and psychological ownership on turnover intention were tested, this section examines whether there are indirect effects of perceived organization support on turnover intention through psychological ownership by adopting the Baron and Kenny (1986) method. Regression results confirmed that v mediates the relationship between the perceived organization support and turnover intention (Sobel test: b = −0.200, *p*-value < 0.05), supporting H_4_; SE mediates the relationship between the perceived organization support and turnover intention (Sobel test: b = −0.111, *p*-value < 0.05), supporting H_4-1_; accountability partially mediates the relationship the between the perception of organizational support and turnover intention (Sobel test: b = −0.073, *p*-value < 0.05), supporting H_4-2_; sense of belonging mediates the relationship between the perceived organization support and turnover intention (Sobel test: b = −0.027, *p*-value < 0.05), supporting H_4-3_; SI mediates the relationship between the organization support and turnover intention (Sobel test b = −0.200, *p*-value < 0.05), supporting H_4-4_.

## 5. Discussion, Implications, and Limitation and Future Works

### 5.1. General Discussion

This study investigated the relationship between organizational support, psychological ownership, and turnover intention. The main findings are as follows: first, there is a significant positive relationship between the perception of organizational support and psychological ownership and each of its dimensions (self-efficacy, taking responsibility, a sense of belonging, and self-identification), which indicates that the higher the degree of psychological ownership of employees in each dimension, the stronger the feeling of organizational support in line with Stinglhamber and Vandenberghe [5] and Daneji and Bambale [53]. This study investigates the overall level of ownership and the components of psychological ownership. The firm can promote employees’ psychological ownership by providing a higher level of organizational support. 

Second, there is a significant negative relationship between organizational support and turnover intention, indicating that the stronger the perception of organizational support, the lower the turnover intention in line with Eisenberger et al. [56] and Lazarova and Caligiuri [60]. This result confirmed the stability of the relationship between perceived organizational support and turnover intention in the context of China. 

Third, there is a significant negative relationship between psychological ownership and turnover intention and also between each dimension of psychological ownership (self-efficacy, taking responsibility, a sense of belonging, and self-identification) and turnover intention, indicating that the higher the degree of psychological ownership of employees, the lower the turnover intention in line with Wagner, Parker, and Christiansen [6]; Olckers and Plessis [74]; and Becker et al. [75].

Finally, psychological ownership and its dimensions mediate the relationship between organizational support and turnover intention. This result provides the main contribution of this study. In addition, this study explored the indirect mechanism of psychological ownership between perceived organizational support and turnover intention. 

### 5.2. Implications

The main findings of this study provided serval implications as follows. First, the findings suggest that increasing organizational support will, in turn, reduce employee turnover intention. The organization’s attitudes toward the organization, which directly influence how well the employee is treated and at work, will also directly determine the employee’s willingness to remain in the organization and strengthen the organization’s development. Therefore, the enterprise needs to ensure the fairness and impartiality of the organization and make employees believe that if they are willing to make efforts, the enterprise will give them corresponding returns. Secondly, the enterprise needs to arrange reasonable jobs and content for employees, provide some opportunities for promotion, and improve salary treatment. Furthermore, there is a need to strengthen the support of supervisors, and to provide necessary tools, guidance, and help, so that employees feel the importance of the enterprise to them. From the establishment of corporate policies and rules to the conduct of daily work to the feedback of questions and recommendations at work, all need to be considered from the employee’s perspective, which creates a supportive organizational atmosphere and improves employee feelings of organizational support.

Secondly, the research results show that increasing employee psychological ownership can reduce employee turnover intention. Therefore, employees’ psychological ownership can be enhanced in the following four ways. (1) Enterprises can help employees make joint career planning, make career development suggestions for employees, and plan future career blueprints to enhance employees’ self-efficacy. (2) Enterprises can provide employees with the opportunity to participate in enterprise management so that employees have the same sense of “master” in the enterprise, create a “home” feeling for the enterprise, and strengthen a sense of belonging of employees to the enterprise. Furthermore, by holding exhibitions and various activities, employees can grasp the status of the enterprise operation and also can participate in the management and management of the enterprise, such as implementing the employee stock ownership plan, understanding the operation mechanism of the enterprise, and allowing employees to participate in the management decision-making of the enterprise as both workers and managers. (3) Enterprises can enable employees to personally supervise various systems in the company, such as the financial system, the promotion system, the reward and punishment system, and the daily norm system of the enterprise, in order to enhance the staff’s sense of responsibility. The firm should also take and implement these opinions and recommendations promptly to promote continuous innovation and rapid growth. At the same time, it may also allow employees to recognize the importance of the recommendations made themselves and the impact on the firm. Employees will also mutually form monitoring mechanisms, which will promote employees to take the responsibilities and risks of the firm actively. (4) Enterprises can establish an excellent corporate culture and increase employees’ self-identity with the enterprise. Enterprises need to have a good reputation image, excellent results and performance, and a high reputation so that employees can regard the enterprise as a symbol of their own identity. Only by improving the employee’s recognition of the enterprise will the employees connect themselves closely with the enterprise, create more positive benefits for the enterprise, and demonstrate their value.

Third, the findings of the study confirm that psychological ownership plays a mediating role between the perception of organizational support and employee turnover intention by showing that when an employee feels the support from the organization, a sense of belonging and recognition of the employee will be strengthened, and a sense of “ownership” to the organization will be stronger and then reduce the employee’s turnover intention. Therefore, while providing organizational support to employees, enterprises can always pay attention to their life and psychological state, such as enhancing enterprise benefits, including salary treatment, insurance benefits, festival gifts, and family arrangements. Increasing the welfare programs of enterprises can attract more and more senior talents and increase the competitive strength of enterprises. Besides paying attention to the working state of employees, the leaders of enterprises and their superiors should also pay attention to their life and psychological state and actively help them solve their work and life problems, such as setting up gymnasium, recreation, and entertainment areas in enterprises; providing dormitories for employees with housing difficulties; providing housing for newlyweds; and launching social activities for young migrant workers in order to solve the problems of marriage. When employees are emotionally and psychologically satisfied, enhanced organizational support can create more benefits for the enterprise.

### 5.3. Limitations and Future Works

Although specific research results have been achieved in this study, it is limited by the capacity, insufficient resources, and the research itself has some deficiencies, as shown in the following aspects. First, the background of the study chose Chinese enterprises, resulting in the sample has some limitations, whether the research findings apply to other countries or organizations needs to be further examined. Future studies are warranted to expand the sample collection and increase the sample size to make the sample more generalizable. Second, the process of action between the perception of organizational support and turnover intention is relatively complex, and its influencing factors should be other variables besides psychological ownership. Future studies should consider incorporating other relevant variables into the research framework to explore the relationship between variables affecting employees’ organizational and psychological aspects of leaving their posts more accurately and comprehensively. Finally, this study did not consider many potential confounder variables, covariates, employees’ health, and other individual factors that affect empirical results. It provides further research opportunities. Further studies should aim to collect individual factors, contextual variables, and emotional variables to extend the proposed model. 

## 6. Conclusions

This study starts with the related theory of organizational support, psychological ownership, and turnover intention to explore the effect of the perceived organizational support on psychological ownership and its various dimensions (self-efficacy, taking responsibility, a sense of belonging, and self-identification); the effect of each dimension of psychological ownership on turnover intention; and the relationship between the perceived organizational support and turnover intention to verify the mediating role of psychological ownership. This study established the relationship model between variables and investigated the relationship between organizational support, psychological ownership, and turnover intention by adopting a survey. The empirical results show that (1) there is a significant positive relationship between the perception of organizational support and psychological ownership and each of its dimensions (self-efficacy, taking responsibility, a sense of belonging, and self-identification), which indicates that the higher the degree of psychological ownership of employees in each dimension, the stronger the feeling of organizational support; (2) there is a significant negative relationship between organizational support and turnover intention, indicating that the stronger the perception of organizational support, the lower the turnover intention; (3) there is a significant negative relationship between psychological ownership and turnover intention, and also between each dimension of psychological ownership (self-efficacy, taking responsibility, a sense of belonging, and self-identification) and turnover intention, indicating that a higher degree of psychological ownership of employees can lower the turnover intention; and (4) psychological ownership and its dimensions (self-efficacy, taking responsibility, a sense of belonging, and self-identification) mediate the relationship between organizational support and turnover intention.

## Figures and Tables

**Table 1:** Measurement design.

Variable	Dimension	Topic Composition	Source
Perceived Organizational Support (POS)	N.A	The organization shows very little concern for me The organization cares about my general satisfaction at work The organization really cares about my well-being The organization strongly considers my goals and values The organization cares about my opinions Even if I did the best job possible, the organization would fail to notice The organization takes pride in my accomplishments at work The organization is willing to extend itself in order to help me perform my job to the best of my ability Help is available from my organization when I have a problem	Wayne, Shore, and Liden [23]
Psychological Ownership (PO)	Self-efficacy	I am confident in my ability to contribute to my organization’s success I am confident I can make a positive difference in this organization I am confident in setting high performance goals in my community	Avey, Avolio, Crossley, and Luthans [39]
	Accountability	I would challenge anyone in my organization if I thought something was done wrong I would not hesitate to tell my organization if I saw something that was done wrong I would challenge the direction of my community to assure it is correct	
	Sense of Belonging	I feel I belong in this organization I am totally comfortable being in this organization This place is home for me	
	Self-identity	I feel this organization’s success is my success I feel being a member in this organization helps define who I am I feel the need to defend my community when it is criticized	
Turnover Intention (TI)	N.A	3.1. I will look for other job opportunities 3.2. I cannot stand the working atmosphere here anymore 3.3. If there is a suitable job opportunity, I will accept it 3.4. I often want to quit my present job	Mobley, Horner, and Hollingsworth [54]
Demographic Variables	N.A	4.1. Gender 4.2. Age 4.3. Education 4.4. Work Experience 4.5. Position	
Firm Information	N.A	Business Type Industry	

**Table 2:** Demographic statistics of the sample (N = 344).

Variable	Category	Frequency	Percentage
Gender	Male	177	51.5
	Female	167	48.5
Age	25 or below	44	12.8
	26–30	133	38.7
	31–35	89	25.9
	36–40	50	14.5
	41–50	25	7.3
	51 or above	3	0.9
Education	High school and below	24	7.0
	College	71	20.6
	Undergraduate	195	56.7
	Master or above	54	15.7
Work Experience	Less than 1 year	39	11.3
	1–5 years	150	43.6
	6–10 years	106	30.8
	11–15 years	39	11.3
	16–20 years	6	1.7
	More than 20 years	4	1.2
Position Level	Ordinary employees	196	57.0
	Frontline managers	80	23.3
	Middle manager	52	15.1
	Senior management	16	4.7
Business Type	State-owned enterprise	31	9.0
	Private enterprise	156	45.3
	Foreign capital and joint ventures	99	28.8
	Others	58	16.9
Industry	Electronic high-tech information	36	10.5
	Machinery manufacturing and steel heavy	26	7.6
	Business services	47	13.7
	Construction and real estate industry	32	9.3
	Financial	28	8.1
	Logistics/sales	42	12.2
	Communication	31	9.0
	Education and sports	56	16.3
	Medical and pharmaceutical chemical	32	9.3
	Agriculture, forestry, animal husbandry, and fishing	4	1.2
	Others	10	2.9

**Table 3:** Descriptive statistics, reliability, and correlation analysis.

	Mean	S.D	α	(1)	(2)	(3)	(4)	(5)	(6)	(7)
SOB (1)	3.15	0.82	0.919	1						
SE (2)	3.65	0.77	0.856	0.387 **	1					
ACC (3)	3.11	0.75	0.882	0.316 **	0.405 **	1				
SoB (4)	3.28	0.91	0.843	0.386 **	0.363 **	0.327 **	1			
SI (5)	3.55	0.87	0.820	0.175 **	0.127 *	0.009	0.146 **	1		
PO (6)	3.42	0.64	0.812	0.487 **	0.724 **	0.668 **	0.720 **	0.485 **	1	
TI (7)	2.64	0.88	0.784	−0.291 **	−0.354 **	−0.291 **	−0.318 **	−0.195 **	−0.446 **	1

Note: Perceived organizational support (POS), self-efficacy (SE), accountability (ACC), sense of belonging (SoB), self-identity (SI), psychological ownership (PO), turnover intention (TI). * *p* < 0.05, ** *p* < 0.01.

**Table 4:** Factor analysis (rotated factor matrix).

Factor						
	1	2	3	4	5	6
POS1	0.731	−0.060	0.083	0.239	0.158	0.069
POS2	0.747	−0.080	0.033	0.199	0.200	−0.034
POS3	0.766	−0.052	0.124	0.144	0.188	−0.035
POS4	0.737	−0.126	0.068	0.215	0.176	0.046
POS5	0.785	−0.041	0.050	0.050	0.131	0.033
POS6	0.786	0.006	0.025	0.100	0.119	0.019
POS7	0.751	−0.146	0.166	−0.032	−0.010	0.155
POS8	0.733	−0.162	0.060	0.057	−0.027	0.102
POS9	0.764	−0.119	0.120	−0.005	−0.019	0.088
SE1	0.190	−0.167	0.180	0.065	0.786	0.084
SE2	0.170	−0.148	0.125	0.160	0.839	0.058
SE3	0.194	−0.146	0.184	0.143	0.818	−0.003
ACC1	0.181	−0.099	0.837	0.101	0.194	−0.028
ACC2	0.180	−0.150	0.837	0.162	0.131	−0.019
ACC3	0.087	−0.103	0.888	0.097	0.143	−0.011
SoB1	0.187	−0.106	0.123	0.854	0.072	0.015
SoB2	0.189	−0.110	0.062	0.810	0.136	0.089
SoB3	0.172	−0.179	0.176	0.788	0.145	0.060
SI1	0.126	−0.109	−0.028	0.076	−0.016	0.805
SI2	0.061	−0.080	0.003	−0.013	0.115	0.861
SI3	0.061	−0.052	−0.021	0.079	0.020	0.868
TI1	−0.177	0.790	−0.093	−0.037	−0.083	−0.033
TI2	−0.088	0.714	−0.065	−0.120	−0.099	−0.073
TI4	−0.081	0.826	−0.033	−0.025	−0.169	−0.100
TI4	−0.114	0.644	−0.164	−0.222	−0.078	−0.064

Note: Perceived organizational support (POS), self-efficacy (SE), accountability (ACC), sense of belonging (SoB), self-identity (SI), psychological ownership (PO), turnover intention (TI).

**Table 5:** Regression results.

	SE	ACC	SoB	SI	PO	TI	TI	TI	TI	TI	TI	TI	TI	TI	TI	TI
POS	0.357 ***	0.295 ***	0.356 ***	0.165 **	0.452 ***	−0.257 ***						−0.161 ***	−0.194 ***	−0.177 ***	−0.234 ***	−0.084
SE							−0.327 ***					−0.268 ***				
ACC								−0.268 ***					−0.211 ***			
SoB									−0.289 ***					−0.223 ***		
SI										−0.177 **					−0.139 **	
PO											−0.421 ***					−0.381 ***
Control Variables	**INCLUDED**															
F	10.273 ***	5.946 ***	10.952 ***	1.812	16.600 ***	6.058 ***	8.168 ***	6.401 ***	6.822 ***	4.376 ***	12.16 ***	8.365 ***	7.35 ***	7.382 ***	6.295 ***	11.106 ***
R²	0.197	0.124	0.207	0.041	0.284	0.126	0.163	0.133	0.140	0.095	0.255	0.184	0.165	0.166	0.145	0.230
Adj R²	0.178	0.103	0.188	0.019	0.267	0.106	0.143	0.112	0.120	0.073	0.207	0.1162	0.143	0.143	0.122	0.210

Note: Perceived organizational support (POS), self-efficacy (SE), accountability (ACC), sense of belonging (SoB), self-identity (SI), psychological ownership (PO), turnover intention (TI). ** *p* < 0.01, *** *p* < 0.001.

## Data Availability

The raw data supporting the conclusions of this article will be made available by the authors, without undue reservation.
